# Genetic differentiation between two varieties of *Oreocharis benthamii* (Gesneriaceae) in sympatric and allopatric regions

**DOI:** 10.1002/ece3.6505

**Published:** 2020-06-28

**Authors:** Qiong Fu, Guo‐Hui Lu, Yu‐Hui Fu, Ying‐Qiang Wang

**Affiliations:** ^1^ Guangdong Provincial Key Laboratory of Biotechnology for Plant Development School of Life Sciences South China Normal University Guangzhou China; ^2^ Guangzhou Key Laboratory of Subtropical Biodiversity and Biomonitoring School of Life Sciences South China Normal University Guangzhou China

**Keywords:** character displacement, genetic diversity, mating system, population structure, reinforcement selection

## Abstract

The pattern of genetic differentiation between diverging species receives much attention as one of the key observable features of speciation. It has often been suggested that introgression between closely related species occurs commonly where their distributions overlap, leading to their becoming more morphologically and genetically similar, but there are a few opposite results. However, most of these studies have been carried out with animals and separate species; few have looked at intraspecific cases, especially in plants. Here, we conduct a comparative study on patterns of genetic differentiation among populations of two varieties of *Oreocharis benthamii* in allopatry and sympatry based on ISSR data for 754 individuals from 26 populations, in order to understand the processes leading to speciation. Contrary to expectations, the facultative xenogamy (mixed mating) species *O. benthamii* has a relatively low genetic diversity within populations (*H* = 0.1014, *I* = 0.1528) and high genetic differentiation among populations (*G*_ST_ = 0.5867, Ф_ST_ = 0.659), as is typically found for selfing species. Genetic variance between the two varieties in sympatric populations (44%, Ф_ST_ = 0.444) is significantly more than that in allopatric populations (14%, Ф_ST_ = 0.138). Consistent with the taxonomical delimitation of the two varieties, all sampled individuals of *O. benthamii* clustered into two genetic groups. Moreover, the genetic structures of populations of both varieties are correlated with their different geographical origins. Our studies show that significant divergence between sympatric populations of the two varieties could be attributed primarily to reinforcement by genetic divergent selection in sympatry where secondary contact had occurred. The major proportion of the genetic variation in outcrossing and mixed mating plants may exist among populations when the populations are distributed in fragmented habitats, due to the paucity of suitable habitat combined with inefficient seed dispersal mechanism and limited pollinator foraging area that may limit the gene flow.

## Graphical abstract

Introgression between closely related species occurs commonly where their distributions overlap, leading to their becoming more morphologically and genetically similar. However, few of these studies have looked at intraspecific cases, especially in plants. Here, we report a reverse situation that the genetic variance between two varieties of *Oreocharis benthamii* in sympatric populations is significantly more than that in allopatric populations.

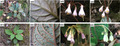

## 1 INTRODUCTION

For a long time, patterns of genetic differentiation between diverging species and the evolution of the mechanisms of speciation isolation have received considerable attention, and the topic has been widely discussed by evolutionary biologists wishing to understand the processes leading to speciation. Genetic differentiation or speciation has mainly occurred during periods when habitats were fragmented and isolated (Bridle, Pedro, & Butlin, 2004), and demographic processes such as long‐distance dispersal are associated with repeated bottlenecks which may have led to increased genetic divergence, due to founder effects and genetic drift (Chen, Liu, Fan, Li, & Liu, 2017; Freedman, Thomassen, Buermann, & Smith, 2010). Increased spatial isolation and decreased population size may lead to the erosion of genetic variation and increased genetic differentiation among populations through genetic drift, increased inbreeding, and reduced gene flow between populations (Honnay, Jacquemyn, Bossuyt, & Hermy, 2005). These evolutionary processes bring about divergence within species and lead to speciation ultimately.

Among many factors affecting speciation, evolutionary theory suggests that natural selection plays a dominant role in speciation. In parapatric and sympatric regions, resource competition and/or reproductive interference between closely related species can be reduced to a minimum due to selection, increasing interspecific divergence and enabling the species to coexist (Pfennig & Pfennig, 2009, 2010). Thus, operational species criteria should reflect different segments of a continuous process of differentiation between evolutionary lineages, resulting in lineage sorting and reciprocal monophyly (Avise, 2000). However, morphologically ambiguous biological species can arise in response to ecological factors (e.g., development of host race specificity by pathogens, adaptation to climatic and geological changes) without observable morphological changes (Kartzinel, Spalink, Waller, & Givnish, 2016). In addition, reproductively isolated cryptic species can arise in populations that are diverging via genetic drift and thus accumulating genetic incompatibilities, independent of natural selection (Nei & Nozawa, 2011). When there is no niche competition in sympatry, reproductive isolation can arise from chromosomal rearrangements in the absence of other barriers (Grant, 1981; Hutchinson, 1957; Levin, 2002). However, cross‐breeding, which can lead to genetic exchange, hybrid formation, and introgression, usually occurs between closely related species in areas where they overlap (sympatry or parapatry), with the result that the two species are genetically more similar in parapatry and sympatry than in allopatry (Anderson & Hubricht, 1938; Mckinnon, Smith, & Potts, 2010; Palme, Su, Palsson, & Lascoux, 2004; Wang, Abbott, Ingvarsson, & Liu, 2014). The potential for hybridization can be reduced by prezygotic or postzygotic mechanisms that constitute interspecies reproductive barriers (Costa, Lambert, Borba, & De Queiroz, 2007; Dobzhansky, 1937; Grant, 1981; Stace, 1989). It has been reported that differing morphology and differentiation of flowering time in the areas of overlap (sympatry or parapatry) between related taxa increases prezygotic isolation with reinforcement; for example, changes in corolla color reduce gamete wastage in *Phlox* (Hopkins & Rausher, 2012; Levin, 1985). Selection for reinforcement might have occurred in parapatry or sympatry leading to increased gene flow barriers, while hybrids arose frequently in the past and were less adaptable than their parent species (Hopkins, 2013). Thus, varieties or incipient species may diverge in sympatry and parapatry, because genetic drift accumulates genetic differences and incompatibilities that lead to the evolution of reproductive barriers when secondary contact takes place (Dobzhansky, 1937; Mayr, 1942; Pfennig & Pfennig, 2009; Rundle & Schluter, 2004). Some studies have shown that when two species overlap geographically, their differentiation is more pronounced in sympatry and diminished or lost entirely in allopatry (Díaz Infante, Lara, Arizmendi, Eguiarte, & Ornelas, 2016 and references therein). A pattern of increased interspecific differentiation between closely related species in sympatry (or parapatry) has been reported for character displacement in a number of animals (Grant & Grant, 2006; Kirschel, Blumstein, & Smith, 2009; Pfennig & Martin, 2010; Pfennig & Murphy, 2002) and plants (Gögler et al., 2015; Grossenbacher & Whittall, 2011; Levin, 1971; Smith & Rausher, 2008; Wang et al., 2014). It is suggested that character displacement may be an alternative to competitive exclusion, arising in sympatry or parapatry so as to decrease competition for resources or bring about reproductive interference (Hopkins, Levin, & Rausher, 2012; Kay & Schemske, 2008; Kirschel et al., 2009; Levin, 1978; van der Niet, Johnson, & Linder, 2006; Urbanelli & Porretta, 2008). However, most of these studies have been carried out with animals and separate species; few have looked at intraspecific cases, especially in plants. In this study, we focus on a morphologically ambiguous plant species, *Oreocharis benthamii* Clarke, which is endemic in South China (Wang, Pan, & Li, 1990; Wang, Pan, Li, Weitzman, & Skog, 1998).

**FIGURE 1:**
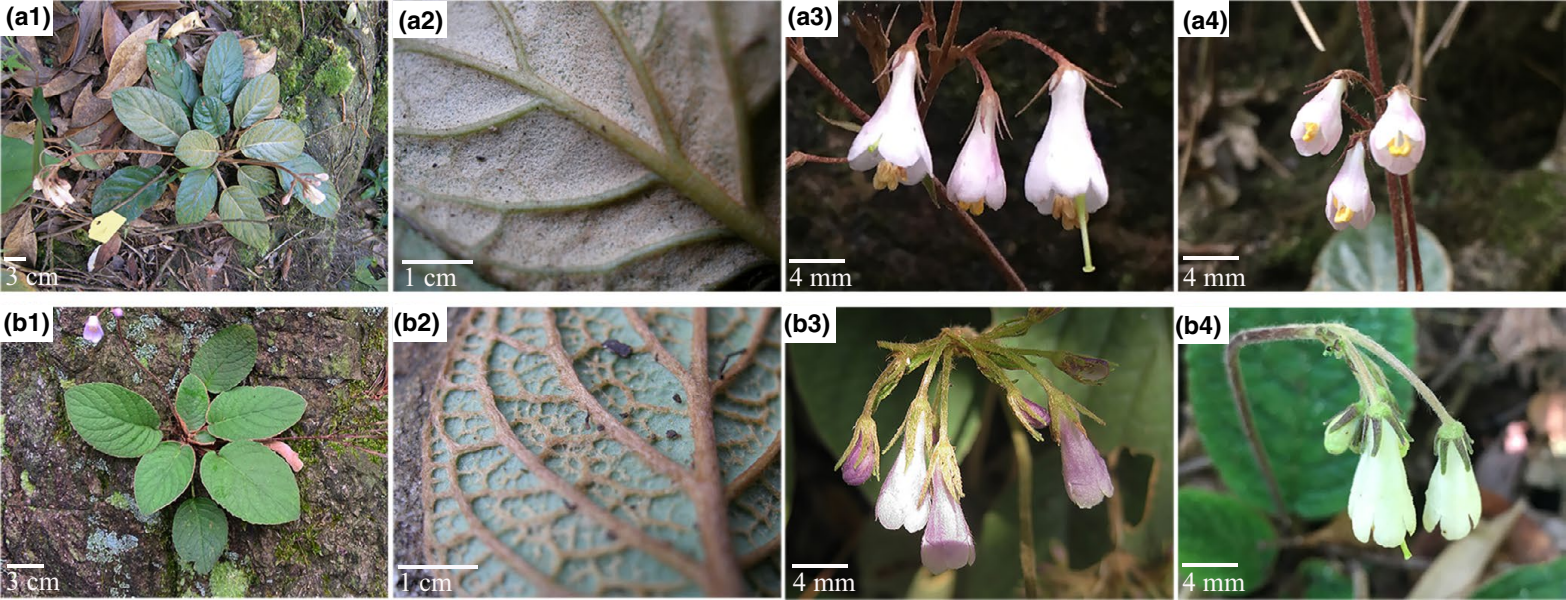
Morphological comparison of *Oreocharis benthamii* var. *benthamii* (a) and var. *reticulata* (b). 1: habit; 2: abaxial leaf surface, showing lateral veins and reticulate veinlets; 3: flowers in allopatry, showing purple corolla; 4: flowers in sympatry (Xiangtou Mountain), showing purple corolla of var. *benthamii* and yellow‐green corolla of var. *reticulata*

**FIGURE 2:**
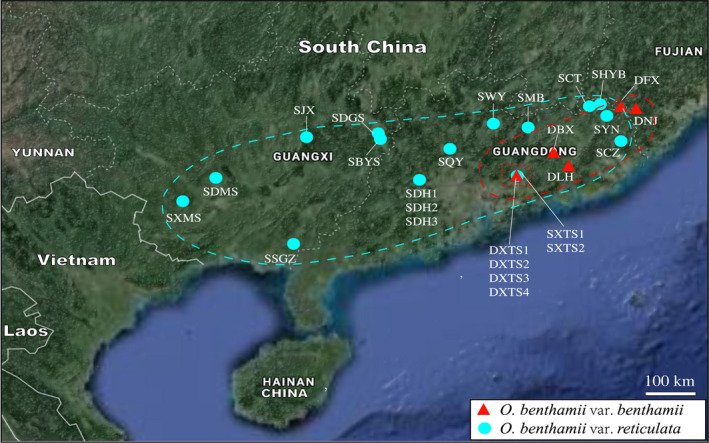
Sampling localities for *Oreocharis benthamii* var. *benthamii* (red triangles) and var. *reticulata* (turquoise circles) in south China. Sympatric area is outlined with a dotted red square. The red and turquoise dashed lines outline the distribution ranges of var. *benthamii* and var. *reticulata*, respectively. The original satellite imagery was obtained from Google Map (Map data 2019 Google; https://maps.google.com/) and modified with Adobe Illustrator CS6 (Adobe Systems Incorporated, San Jose, CA, USA)

**FIGURE 3:**
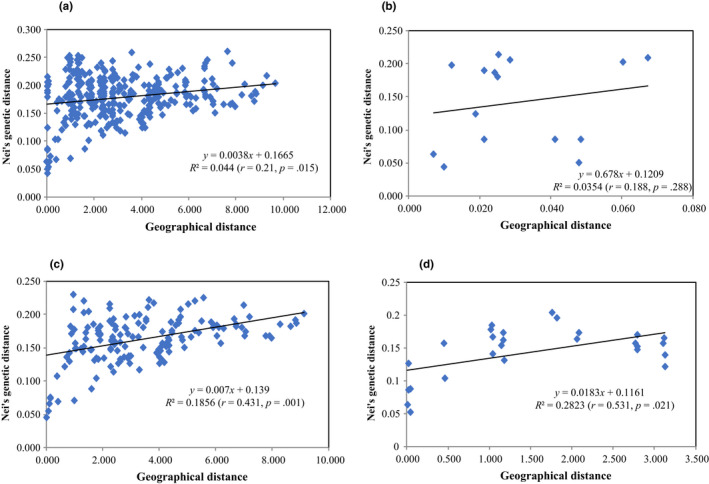
Plot of Mantel test showing the relationships of genetic and geographic distances in populations of *Oreocharis benthamii* from (a) the entire distribution area, (b) the sympatric region, and for populations of (c) var. *reticulata* and (d) var. *benthamii*

**FIGURE 4:**
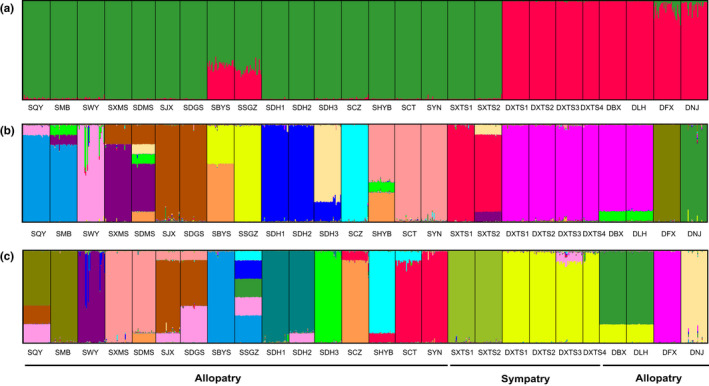
Genetic group structure shown by STRUCTURE analysis for 26 populations of *Oreocharis benthamii* (*K* = 2, 15, 17). Each individual vertical bar represents one population and different colors represent different gene pools

Plants of *O. benthamii* are perennial herbs comprising two varieties, var. *benthamii* and var. *reticulata* Dunn, with no obvious morphological differences (Figure 1). The minor distinctions between them are that the leaf blade of var. *reticulata* is ovate‐orbicular (vs. var. *benthamii* is oblong to ovate), and its lateral veins and reticulate veinlets are more prominent than those of var. *benthamii* (Li & Wang, 2004; Wang et al., 1998). However, these distinctions are not always obvious, especially in leaf blade shape and when the lateral veins and reticulate veinlets are covered with the densely woolly on leaves in some populations, resulting in mistaken identification. Our unpublished phylogenetic analysis of the enlarged *Oreocharis* species basing on *trn*L‐F and ITS sequence variation showed that var. *benthamii* and var. *reticulata* formed a monophyletic clade and were genetically closely related. Both are small herbs and occur on rock walls in valley or moist soil in humid monsoon forests, and their distribution ranges are substantially the same (Li & Wang, 2004; Wang et al., 1998). However, the two varieties rarely overlap at the same site, and no mixed populations have been found. Our field observations showed that flowers of both varieties were generally purple to blue in allopatry (Figure 1a‐3,b‐3), but flowers of var. *reticulata* in sympatric populations were yellow‐green (Figure 1b‐4) and those of var. *benthamii* were purple (Figure 1a‐4). The two varieties, both of which are facultative xenogamy (mixed mating), exhibit partial overlap of flowering periods, sharing of pollinators, similarities between floral events such as stigma receptivity and pollen release, similar floral morphology, and partial compatibility in intervarietal crosses (Guo, 2011). Sympatric and allopatric populations of the two varieties of *O. benthamii* represent a good system with which to investigate the erosion of genetic variation and increased genetic differentiation among populations through genetic drift, increased inbreeding, and reduced gene flow between populations and thus to understand the processes leading to speciation and the maintenance of species (variety) boundaries. The ISSR (inter simple sequence repeat) is an easy handling, good reproducibility, low cost, quick and highly informative technique, and widely used for plant population genetic studies (Sharma, Sharma, Rana, & Chahota, 2015; Tabina et al., 2016; Zietkiewicz, Rafalski, & Labuda, 1994), in spite of the incapacity to distinguish heterozygous allele states in an individual. Here, we assess the genetic diversity and population structure of *O*. *benthamii* using ISSR markers, focusing on the following questions: (1) How genetic diversity and genetic differentiation are distributed throughout all populations of the species; (2) whether there is increased or reduced genetic differentiation between sympatric populations compared with allopatric populations of these two varieties.

## 2 MATERIALS AND METHODS

### 2.1 Material sampled

Both *O. benthamii* var. *benthamii* and var. *reticulata* are nonclonal perennial acaulescent herbs endemic to China, and their distribution ranges are partially overlapped, mainly in southern China (Li & Wang, 2004; Wang et al., 1998). However, during our 12 years (2007–2019) of fieldwork, we only found the two varieties coexisted at one site and var. *benthamii* only distributed at the regions of eastern and central Guangdong, northern Guangdong, and western Fujian (Figure 2, Table S1). In this study, the areas of distribution of *O. benthamii* were divided into sympatric (where the populations of the two varieties occurred within ca. 1–5 km at the same site, within the potentially foraging distance of the bee pollinators and the limited range of the seed dispersal) and allopatric (where the populations of the two varieties were separated by over 10 km, without opportunity for exchange of genetic material) (Figure 2), to investigate patterns of genetic differentiation and genetic structure. Leaf tissue was collected from 754 plants representing 26 extant populations at one sympatric and 20 allopatric localities across the distributional ranges of these two varieties (Table S1, Figure 2). Samples were taken from 18 to 30 plants at least 2–3 m apart in each population. In total, 16 populations of var. *reticulata* and 4 populations of var. *benthamii* were sampled from allopatric areas, while 2 populations of var. *reticulata* and 4 populations of var. *benthamii* were sampled from the sympatric area. Fresh leaflets were dried with silica gel and stored at −80°C until required for DNA extraction. Voucher specimens were prepared from most of the populations and deposited at the herbarium of South China Normal University (SN).

### 2.2 DNA extraction and ISSR PCR reactions

Total genomic DNA was extracted from silica‐dried leaf material using a modified cetyl trimethyl ammonium bromide (CTAB) procedure (Doyle & Doyle, 1987). The quality and concentration of the extracted DNA were estimated on a 0.8% agarose gel and a Nano‐100 spectrophotometer (Allsheng). Nuclear DNA was then PCR amplified using ISSR primers obtained from the University of British Columbia. Initially, 100 ISSR primers were screened in 18 samples from six populations of the two varieties and ten polymorphic primers (808, 834, 841, 847, 857, 873, 879, 881, 899, and 900) were eventually selected for generating ISSR profiles. Reactions were performed in a total volume of 20 μl containing 2.0 μl 10× PCR buffer, 2.0 mM MgCl_2_, 0.7 mM dNTPs, 2.0 μM primer, 2.0 units of Taq polymerase, and 30 ng of template DNA and double‐distilled water. Polymerase chain reactions (PCRs) amplification was conducted on a Bio‐Rad T100™ Thermal Cycler (Bio‐Rad) under the following conditions: initial denaturation at 94°C for 5 min, followed by 39 cycles of 30 s at 94°C, 45 s at a primer‐specific annealing temperature, extension for 90 s at 72°C, and a 10‐min final extension step at 72°C. The annealing temperature for each primer is given in Table S2. Negative controls, which lacked template DNA in PCR, were included to test for possible contamination. To ensure reproducibility of the amplification, duplicate PCR amplifications were performed and only clear and reproducible bands were scored. Amplification products were electrophoretically separated in 1.8% agarose gels, together with a 100 bp ladder as a size marker, and visualized on a UV transilluminator of the gel documentation system (Bio‐Rad Gel Doc XR+, America). The images of the gels were analyzed using Image Lab software (Bio‐Rad) to score for the presence or absence of bands and to assign a fragment size to each band. The presence or absence of bands was also visually confirmed.

### 2.3 Data analysis

All clear and reproducible amplified fragments were scored as binary characters (presence or absence) and converted into a binary data matrix. The resulting presence/absence data matrix was analyzed using POPGENE version 1.32 (Yeh, Yang, & Boyle, 1999) to estimate the following genetic diversity parameters based on individual genotypes for each population: number of observed alleles (*N*a), number of effective alleles (*N*e), percentage of polymorphic loci (PPL), Nei's genetic diversity (*H*), and Shannon's information index (*I*). At the species level, three genetic diversity measures (*H*_T_: total genetic diversity; *H*s: genetic diversity within populations; *and G*_ST_: relative magnitude of genetic differentiation among populations *G*_ST_ = (*H*_T_ − *H*s)/*H*_T_)), the level of gene flow (*N*m), and the genetic distance between populations were also computed using the model presented in Nei (Nei, 1972, 1973).

To visualize the genetic relationships among individuals and populations of var. *reticulata* and var. *benthamii*, we constructed a neighbor‐joining (NJ) tree based on Nei's genetic distance with the program NEIGHBOR incorporated in the software package PHYLIP version 3.6 (Felsenstein, 2005). Bootstrap support for internal nodes was estimated with a 1,000 distance matrix of replicates generated in AFLP‐SURV version 1.0 (Vekemans, Beauwens, Lemaire, & Roldán‐Ruiz, 2002), and a bootstrap consensus tree was generated with Consense in PHYLIP. Based on Nei's similarity coefficient, an unweighted pair‐group method arithmetic mean (UPGMA) dendrogram of pairwise population genetic identity was also generated using NTSYS 2.1. Principal coordinate analysis (PCoA) based on the Nei's genetic distance in GenAlEx ver. 6.5 (Peakall & Smouse, 2012) was used to demonstrate the relative genetic distances within and among populations. A Bayesian model‐based clustering method implemented in STRUCTURE ver. 2.2 (Pritchard, Stephens, & Donnelly, 2000) was used to infer the number of genetic units and their spatial delimitation for 26 populations of var. *reticulata* and var. *benthamii*. Ten independent runs were performed for each *K* (the number of clusters), from *K* = 1 to 26. The maximum number of clusters used was greater than the number of populations in order to detect possible substructuring within the samples. All runs were performed with the admixture model without prior population information, assuming correlated allele frequencies among populations, with burn‐in and run lengths of 100,000 and 1,000,000 iterations, respectively. The optimal number of clusters (*K*) was determined following the guidelines from Wang (2019), and the results across the independent analyses were combined by the program Clumpp (Jakobsson & Rosenberg, 2007) and visualized with Distruct v2.1 (Rosenberg, 2004).

Hierarchical structure of genetic variation and pairwise genetic distance (Φ_ST_) (Excoffier, Smouse, & Quattro, 1992; Meirmans, 2006) among the populations was determined by analysis of molecular variance (AMOVA) with GenAlEx ver.6.5. Significance levels of the variance components were based on 999 permutations. Mantel tests were performed to analyze the effects of geographical distance on genetic variation.

## 3 RESULTS

### 3.1 ISSR polymorphism and genetic diversity

Values obtained for ISSR polymorphism and genetic diversity are summarized in Table S2 and Table 1. The ten primers produced 454 reproducible ISSR bands (an average of 45.4 bands per primer) from the 26 populations of *O. benthamii*, and the size of the bands ranged from 120 to 2,200 bp. At the species level, there were 454 (100%) polymorphic ISSR bands, of which 435 (95.81%) and 323 (71.15%) polymorphic bands were identified in the populations of var. *reticulata* and var. *benthamii,* respectively. Of the 454 bands, 131 (28.54%) were found only in the populations of var. *reticulata* and 19 (4.19%) were present only in var. *benthamii*. The populations of the species showed an overall Shannon diversity index (*I*) of 0.3822. Shannon's diversity index of var. *reticulata* (*I = *0.3698) was greater than that of var. *benthamii* (*I* = 0.2977). At the population level, the percentage of polymorphic loci (PPL) and Shannon's diversity index (*I*) of the species was, respectively, 30.34% and 0.1528 on average. The average level of genetic diversity of var. *reticulata* populations (PPL = 32.47%, *I* = 0.1641) was significantly higher than that of var. *benthamii* (PPL = 25.55%, *I* = 0.1272). Populations SDH3 (var. *reticulata*) and DXTS3 (var. *benthamii*) showed the highest levels of genetic diversity (PPL = 40.53%, *I* = 0.2252; PPL = 30.18%, *I* = 0.1536, respectively), while populations SQY (var. *reticulata*; PPL = 25.99%, *I* = 0.1353) and DBX (var. *benthamii*; PPL = 20.26%, *I* = 0.1052) exhibited the lowest levels.

**TABLE 1:** Genetic variability parameters based on ISSR analysis for *Oreocharis benthamii* var. *reticulata* and var. *benthamii*

Taxon	Population	*N*a	*N*e	*H*	*I*	PL	PPL (%)
var. *reticulata*	SDH1	1.3348	1.1921	0.1130	0.1699	152	33.48
	SDH2	1.3348	1.1963	0.1154	0.1731	152	33.48
	SDH3	1.4053	1.2671	0.1526	0.2252	184	40.53
	SHYB	1.3789	1.2374	0.1365	0.2025	172	37.89
	SCT	1.3546	1.2089	0.1217	0.1824	161	35.46
	SYN	1.3128	1.1697	0.1012	0.1535	142	31.28
	SXTS1	1.2930	1.1695	0.0989	0.1482	133	29.30
	SXTS2	1.2731	1.1543	0.0907	0.1365	124	27.31
	SXMS	1.3084	1.1706	0.1004	0.1514	140	30.84
	SDMS	1.2974	1.1809	0.1050	0.1566	135	29.74
	SJX	1.3348	1.1852	0.1097	0.1658	152	33.48
	SDGS	1.3062	1.1485	0.0907	0.1397	139	30.62
	SSGZ	1.3062	1.1590	0.0953	0.1451	139	30.62
	SBYC	1.3084	1.1574	0.0951	0.1456	140	30.84
	SQY	1.2599	1.1532	0.0902	0.1353	118	25.99
	SMB	1.3216	1.1738	0.1043	0.1586	146	32.16
	SCZ	1.3172	1.1765	0.1049	0.1585	144	31.72
	SWY	1.3965	1.2348	0.1376	0.2063	180	39.65
	Mean	1.3247	1.1853	0.1091	0.1641	147.4	32.47
	Total	1.9581	1.3882	0.2372	0.3698	435	95.81
var. *benthamii*	DXTS1	1.2489	1.1387	0.0828	0.1252	113	24.89
	DXTS2	1.2753	1.1491	0.0898	0.1363	125	27.53
	DXTS3	1.3018	1.1714	0.1019	0.1536	137	30.18
	DXTS4	1.2511	1.1371	0.0811	0.1229	114	25.11
	DFX	1.2797	1.1471	0.0878	0.1337	127	27.97
	DNJ	1.2643	1.1371	0.0814	0.1239	120	26.43
	DLH	1.2203	1.1344	0.0783	0.1167	100	22.03
	DBX	1.2026	1.1172	0.0699	0.1052	92	20.26
	Mean	1.2555	1.1415	0.0841	0.1272	116.0	25.55
	Total	1.7115	1.3248	0.1939	0.2977	323	71.15
Species	Mean	1.3034	1.1718	0.1014	0.1528	137.7	30.34
	Total	2.0000	1.4057	0.2459	0.3822	454	100.00

Abbreviations: *H*, Nei's gene diversity; *I*, Shannon's information index; *N*a, number of observed alleles; *N*e, number of effective alleles; PL, number of polymorphic loci; PPL: percentage of polymorphic loci.

### 3.2 Genetic differentiation and gene flow

Genetic differentiation statistics for all populations of *O. benthamii* are presented in Table 2. The mean Nei's *G*_ST_ for all populations of the species was estimated as 0.5867, indicating that 58.67% of the genetic variability was distributed among populations, and the estimate of gene flow (*N*m) per generation among populations was 0.3522. Genetic differentiation among populations in var. *reticulata* (*G*_ST_ = 0.5394) was slightly lower than that in var. *benthamii* (*G*_ST_ = 0.5614); accordingly, the mean estimated gene flow among populations in var. *reticulata* (*N*m = 0.4269) is a little higher than that in var. *benthamii* (*N*m = 0.3906). The AMOVA analysis (Table 3) was consistent with Nei's genetic differentiation statistics (Table 2), showing that 66% (Φ_ST_ = 0.659) of the total variation in the species was partitioned among populations, and the variances among populations in var. *reticulata* (62%, Φ_ST_ = 0.625) and var. *benthamii* (65%, Φ_ST_ = 0.650) were almost the same. Of the total molecular variance, 16% was attributable to the divergence between var. *reticulata* and var. *benthamii*, 53% to population differences within varieties, and 31% to individual differences within populations (Table 4). At the species level, the variance (64%, Ф_ST_ = 0.639, *G*_ST_ = 0.5267) among sympatric populations was almost the same as that (65%, Ф_ST_ = 0.648, *G*_ST_ = 0.5711) among allopatric populations (Tables 2 and 3). However, the divergences among allopatric populations of both varieties were significantly greater than that among sympatric populations (var. *reticulata*: *G*_ST_ = 0.5285 vs. 0.1757, Ф_ST_ = 0.616 vs. 0.360; var. *benthamii*: *G*_ST_ = 0.5736 vs. 0.3852, Ф_ST_ = 0.690 vs. 0.516). In addition, the variance (44%, Ф_ST_ = 0.444) between the two varieties in sympatric populations was significantly more than that (14%, Ф_ST_ = 0.138) in allopatric populations (Table 4).

**TABLE 2:** Statistics for genetic differentiation among populations of *Oreocharis benthamii*

Taxa	*H*_T_	*H*_S_	*G*_ST_	*N*m
Species				
Allopatric populations	0.2437	0.1045	0.5711	0.3755
Sympatric populations	0.1920	0.0909	0.5267	0.4493
Total	0.2453	0.1014	0.5867	0.3522
var. *reticulata*				
Allopatric populations	0.2351	0.1109	0.5285	0.4460
Sympatric populations	0.1150	0.0948	0.1757	2.3451
Total	0.2368	0.1091	0.5394	0.4269
var. *benthamii*				
Allopatric populations	0.1860	0.0793	0.5736	0.3717
Sympatric populations	0.1446	0.0889	0.3852	0.7981
Total	0.1918	0.0841	0.5614	0.3906

Abbreviations: *G*_ST_, coefficient of gene differentiation among populations; *H*_S_, average within‐population diversity; *H*_T_, total population diversity; *N*m, estimate of gene flow from *G*_ST_.

**TABLE 3:** Summary of molecular variance analysis (AMOVA) of *Oreocharis benthamii* var. *reticulata* and var. *benthamii*

Taxa	Regions	Source of variation	*df*	SS	Est. var.	Variation (%)	Ф_ST_	*p*
Species	Entire distribution area	Among populations	25	30,708.004	41.625	66%	0.659	.001
		Within populations	728	15,669.747	21.524	34%		
		Total	753	46,377.751	63.150	100%		
	Sympatry	Among populations	5	4,697.295	33.246	64%	0.639	.001
		Within populations	161	3,021.878	18.769	36%		
		Total	166	7,719.174	52.015	100%		
	Allopatry	Among populations	19	23,363.950	41.140	65%	0.648	.001
		Within populations	567	12,647.869	22.307	35%		
		Total	586	36,011.819	63.447	100%		
var. *benthamii*	Entire distribution area	Among populations	7	6,473.997	32.211	65%	0.650	.001
		Within populations	218	3,788.472	17.378	35%		
		Total	225	10,262.469	49.589	100%		
	Sympatry	Among populations	3	1,619.970	19.732	52%	0.516	.001
		Within populations	103	1,904.011	18.486	48%		
		Total	106	3,523.981	38.217	100%		
	Allopatry	Among populations	3	3,300.195	36.429	69%	0.690	.001
		Within populations	115	1,884.461	16.387	31%		
		Total	118	5,184.655	52.815	100%		
var. *reticulata*	Entire distribution area	Among populations	17	19,746.663	38.808	62%	0.625	.001
		Within populations	510	11,881.275	23.297	38%		
		Total	527	31,627.938	62.105	100%		
	Sympatry	Among populations	1	344.333	10.835	36%	0.360	.001
		Within populations	58	1,117.867	19.274	64%		
		Total	59	1,462.200	30.109	100%		
	Allopatry	Among populations	15	17,085.675	38.132	62%	0.616	.001
		Within populations	452	10,763.408	23.813	38%		
		Total	467	27,849.083	61.945	100%		

Abbreviations: *df*, degrees of freedom; Est. var., estimated variance; *p*, probability of null hypothesis; SS, sum of squares; Ф_ST_, statistics analogy to *F*_ST_ statistics.

**TABLE 4:** Molecular variance components analysis (AMOVA) for *Oreocharis benthamii*

Regions	Source of variation	*df*	SS	Est. var.	Variation (%)	Ф_ST_	*p*
Entire distribution area	Between taxa (two varieties)	1	4,487.344	10.729	16%	0.155	.001
	Among populations	24	26,220.659	36.940	53%	0.632	.001
	Within populations	728	15,669.747	21.524	31%		
	Total	753	46,377.751	69.193	100%		
Sympatry	Between taxa (two varieties)	1	2,732.992	28.742	44%	0.444	.001
	Among populations	4	1,964.303	17.286	27%	0.479	.001
	Within populations	161	3,021.878	18.769	29%		
	Total	166	7,719.174	64.798	100%		
Allopatry	Between taxa (two varieties)	1	2,978.081	9.660	14%	0.138	.001
	Among populations	18	20,385.870	37.853	54%	0.629	.001
	Within populations	567	12,647.869	22.307	32%		
	Total	586	36,011.819	69.819	100%		

Abbreviations: *df*, degrees of freedom; Est. var., estimated variance; *p*, probability of null hypothesis; SS, sum of squares; Ф_ST_, statistics analogy to *F*_ST_ statistics.

The Mantel test (Figure 3) revealed that the genetic divergence of all populations of *O. benthamii* from across the entire distribution area was not significantly correlated with geographic distance (*r* = .21, *p* = .015). In addition, there was no correlation between genetic and geographical distances among all sample populations of *O. benthamii* from the sympatric region (Xiangtou Mountain) (*r* = .188, *p* = .288). However, there was a weak or moderate relationship among populations within var. *reticulata* (*r* = .431, *p* = .001) or var. *benthamii* (*r* = .531, *p* = .021).

### 3.3 Genetic structure and cluster analysis

Bayesian genetic STRUCTURE analyses revealed that the log likelihood reached a maximum value at *K* = 2 and assigned all populations of the species to one of two genetic clusters based on allele frequencies (Figure 4a; Table S3), in which all individuals were assigned to the same genetic cluster within populations except for two populations of var. *reticulata* (SSGZ, SBYS) and two populations of var. *benthamii* (DFX, DNJ), which suggests a higher level of admixture of the two gene pools within the four populations. With *K* = 15 (the best *K* based on Parsimony Index) and 17 (the best *K* based on Pr[X|K] value), about half of the populations of both varieties were assigned to independent genetic clusters, and some geographically close populations were placed into the same genetic clusters (Figure 4b,c; Table S3). In other populations, there was some degree of admixture of different gene pools in individuals within each population.

**FIGURE 5:**
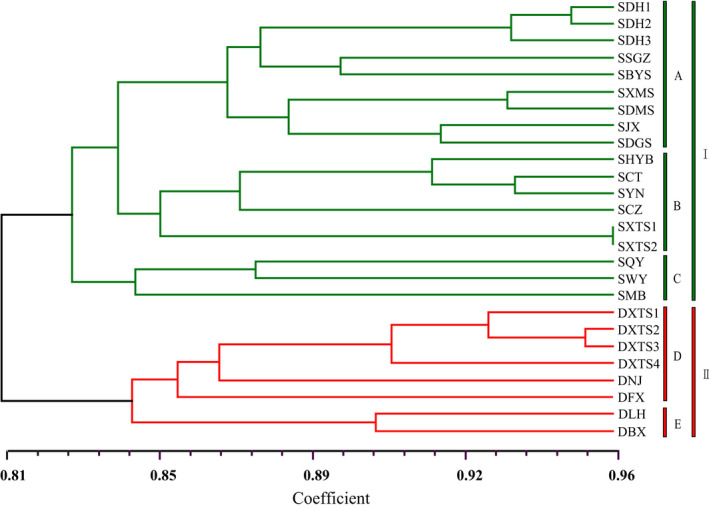
UPGMA dendrogram based on Nei's genetic identity for 26 populations of *Oreocharis benthamii* var. *reticulata* (green) and var. *benthamii* (red)

The UPGMA dendrogram based on Nei's similarity coefficient grouped the 26 populations of the species into two clusters with a similarity index value of 0.81 (Figure 5). In the dendrogram, all the populations from each variety clustered together, as cluster I (var. *reticulata*) (highlighted green) and cluster II (var. *benthamii*) (highlighted red). Cluster I further formed two groups with three well‐resolved clades (A, B, C). One group consisted of two clades (A and B), and the other group consisted only of clade C, which comprised all populations of var. *reticulata* from northern Guangdong. Clade A comprised all populations of var. *reticulata* from western Guangdong and Guangxi, whereas clade B included all populations from eastern and central Guangdong. Cluster II also further formed two well‐resolved clades (D, E). Clade D comprised all populations of var. *benthamii* from central and northeast Guangdong and Fujian, and clade E consisted of all populations from southeast Guangdong. An unrooted neighbor‐joining (NJ) tree based on Nei's genetic distance also separated all sampled individuals into two clusters in accordance with the morphological delimitation between var. *reticulata* (highlighted green) and var. *benthamii* (highlighted red) (Figure S1). The NJ tree also showed that the divergence of populations of var. *reticulata* could be attributed to their different geographical origins, the regions of eastern and central Guangdong, northern Guangdong, and western Guangdong and Guangxi. However, the clade of populations in eastern and central Guangdong and the clade of populations in northern Guangdong clustered together (but only at a low similarity index value of 345/1,000), a result different from that in the UPGMA dendrogram. The var. *benthamii* cluster formed two groups with three well‐resolved branches. One group consisted only of population DFX from northeast Guangdong, and the other group consisted of two branches, population DNJ from Fujian and another clade including all populations from eastern and central Guangdong. The STRUCTURE analysis (Figure 4) revealed a pattern that was consistent with the NJ tree.

The PCoA analysis (Figure S2) shows two main groupings, var. *reticulata* (ringed in green) and var. *benthamii* (red). The var. *reticulata* group was divided into three subsets, which comprised all populations from, respectively, eastern and central Guangdong, northern Guangdong, and western Guangdong and Guangxi. There were also three subsets in the var. *benthamii* group: DFX, DNJ, and the subset from eastern and central Guangdong. The result of PCoA analysis thus confirmed the partitioning results obtained from UPGMA clustering and the NJ tree, especially the latter.

## 4 DISCUSSION

### 4.1 Do facultative xenogamy plants have high genetic diversity within populations and low differentiation among populations, as is typically found for outcrossing species?

Outcrossing species would be expected to have higher within‐population genetic diversity and lower population differentiation values than selfing species (Clasen, Moss, Chandler, & Smith, 2011; Hamrick & Godt, 1989; Nybom, 2004). Most facultative xenogamy (mixed mating) plants should be more similar to outcrossing species, since they have a preference for outcrossing pollination compared to selfing pollination. However, our results reveal that the mixed mating *O. benthamii*, whether at the species or at the variety level, has a very low level of population genetic diversity and a high level of population differentiation, but a high level of species and variety genetic diversity.

The high level of population differentiation and low level of genetic diversity within populations of *O. benthamii* may be attributed to its habitat, the short distances over which pollen travels and the restricted seed dispersal. *Oreocharis benthamii* grows widely on rocky outcrops in subtropical humid monsoon forests, valleys, and cliffs. These harsh habitats are usually isolated and distributed in a mosaic pattern of mixed bare areas and forests with acidic soils. This mosaic distribution pattern would have presented a significant barrier to gene flow and the spread of the species and would have caused habitat fragmentation and a high degree of population isolation. Previous studies have shown that an increase in spatial isolation and decrease in population size may lead to the impairment of genetic diversity within populations and an increase in genetic differentiation among populations through genetic drift, inbreeding and reduction in gene flow between populations (Bentley, Barker, & Dold, 2015; Buza, Young, & Thrall, 2000; Ellstrand & Elam, 1993; Honnay et al., 2005; Toczydlowski & Waller, 2019; Young, Boyle, & Brown, 1996). Gene flow between plant populations occurs mostly via pollen movement and seed dispersal. The estimate of gene flow (*Nm* = 0.3522 on average), which was <1, for *O. benthamii* suggested that gene flow between populations of *O. benthamii* was limited by the extent of pollen and seed dispersal and was insufficient to counter the effects of random drift (Han et al., 2009; Real, 1994). Because it is pollinated by parasitic bees (Guo, 2011), the short flight ranges of the insects limit pollen dispersal in *O. benthamii*. This mosaic distribution pattern also restricts seed dispersal by wind and gravity, due to physical barriers and limited seed dispersal ability. Moreover, both the two varieties have a delayed autogamy mechanism and there is a dearth of effective pollinators for the populations (Guo, 2011), like as *Oreocharis synergia* (Chen, Möller, Chen, Rui, & Shui, 2015) and *Oreocharis ninglangensis* (Chen, Chen, Möller, Wen, & Shui, 2016). Our results also show that the highest percentage of genetic variation (66%) existed among populations, while only 34% of the genetic variance resided within different populations in *O. benthamii*. This implies that the major proportion of genetic variation in outcrossing and mixed mating (facultative xenogamy) plants may exist among populations, rather than within populations, when the plants are distributed in fragmented habitats, due to the paucity of suitable habitat combined with inefficient seed dispersal mechanism (e.g., no appendages to aid wind dispersal, Kokubugata, Hirayama, Peng, Yokota, & Möller, 2011) and limited pollinator foraging area that may limit the gene flow.

### 4.2 Genetic differentiation in *O. benthamii*

The results of both NJ analysis and UPGMA clustering of all individuals sampled from *O. benthamii* were completely congruent with the morphological delimitation between var. *reticulata* and var. *benthamii*. Bayesian genetic structure analysis (*K* = 2) also detected two gene pools (var. *reticulata* and var. *benthamii*) for all populations of *O. benthamii*, confirming the partitioning results revealed by UPGMA clustering and the NJ tree. All these approaches demonstrated that *O. benthamii* could be differentiated into two distinct varieties. There were different admixtures within individuals in all populations except for two populations (SDHS2 and SXMS) of var. *reticulata* from west Guangdong and Guangxi and one population (DBX) of var. *benthamii* from eastern Guangdong. Admixed individuals, reflecting introgression, were detected in both directions, and the gene flow between the two gene pools (var. *reticulata* and var. *benthamii*) was significantly asymmetric in most populations. The small amount of admixture within individuals of most populations probably arose from gene flow between var. *reticulata* and var. *benthamii* in processes associated with recent migration in the regions of overlap as well as earlier introgression.

### 4.3 Increased genetic divergence between the two varieties of *O. benthamii* in the sympatric region

It has been suggested that closely related taxa may be more similar in sympatry and parapatry than in allopatry, because introgression often occurs between closely related taxa in areas of overlap (e.g., Anderson & Hubricht, 1938; Behm, Ives, & Boughman, 2010; Mckinnon et al., 2010; Mehner et al., 2010; Palme et al., 2004; Rieseberg & Wendel, 1993; Sullivan, Lavoue, Arnegard, & Hopkins, 2004). However, this is not likely to be the case for var. *reticulata* and var. *benthamii*. The results presented here show that differentiation among populations of *O. benthamii* in the allopatric region (*G*_ST_ = 0.5711, Ф_ST_ = 0.648, 65%) is similar to that in the sympatric region (*G*_ST_ = 0.5267, Ф_ST_ = 0.639, 64%). However, the variance between the two varieties in sympatric populations (44%, Ф_ST_ = 0.444) is significantly greater than that in allopatric populations (14%, Ф_ST_ = 0.138). This indicates that the degree of differentiation between the two varieties in the sympatric region is significantly higher than that in the allopatric region. A high level of differentiation between species in sympatry or parapatry may arise from demographic processes associated with range expansions by the two species (Freedman et al., 2010). Under conditions in which sympatric or parapatric populations of two species originate from allopatric ones, repeated bottleneck events associated with recent range expansion may result in an increase in genetic differentiation between their populations in sympatry or parapatry due to founder events and genetic drift (Wang et al., 2014). In addition, selection can act to minimize resource competition or reproductive interference between closely related species in parapatry or sympatry, thereby increasing interspecific differentiation and thus enabling the species to coexist (Pfennig & Pfennig, 2010). These factors may apply in the cases of var. *reticulata* and var. *benthamii*. However, strong genetic drift associated with demographic expansion would result in reduced genetic diversity (Allendorf, 1986; Ellstrand & Elam, 1993), which was evident from comparisons of Nei's genetic diversity (*H*) made between allopatric and sympatric populations of var. *reticulata*, but not those of var. *benthamii* (Table 2). This suggests that sympatric populations probably represent zones of secondary contact between the two varieties of *O. benthamii*, like as that has been shown in *Oreocharis* × *heterandra* (Puglisi, Wei, Nishii, & Möller, 2011) and that var. *reticulata* was subjected to demographic expansion events, but var. *benthamii* was not. Moreover, only a weak geographical pattern of isolation by distance was found when intervarietal and intravarietal comparisons were made across the full distribution area, while no geographical pattern of isolation was found in sympatry (Figure 3). Furthermore, no geographical barriers to gene flow between allopatric and sympatric populations of either of the two varieties were found, indicating that geographical distance may has not played a major role, in restricting gene flow within and between var. *reticulata* and var. *benthamii* in the sympatry. Genetic drift or demographic processes are therefore unlikely to have caused primarily the increased genetic differentiation between the two varieties in sympatry. Thus, it is likely that divergent selection (Brown & Wilson, 1956; Pfennig & Pfennig, 2009) may have made a major contribution to the increased genetic differentiation between var. *reticulata* and var. *benthamii* in sympatry, just as was found to be the case in two closely related fir species, *Abies chensiensis* and *Abies fargesii* (Wang et al., 2014).

Selection may increase ecological adaptation to different habitats, resulting in a decrease in interspecific competition in sympatry or parapatry (Nosil, 2012; Schluter, 2001). Our field observations showed that flowers of both var. *reticulata* and var. *benthamii* were generally purple to blue (Figure 1a‐3,b‐3), but that all flowers of var. *reticulata* in sympatric populations (Xiangtou mountain) were yellow‐green (Figure 1b‐4), that is, there had been reproductive character displacement (Brown & Wilson, 1956; Pfennig & Pfennig, 2009). Character displacement can arise during the speciation process (reinforcement selection) or as a way of reducing gamete wastage after speciation (Butlin, 1987; Higgie, Chenoweth, & Blows, 2000; Hopkins, 2013; Jang & Gerhardt, 2006; Kay & Schemske, 2008; Liou & Price, 1994; Noor, 1995; Nosil, Crespi, & Sandoval, 2003; Ortiz‐Barrientos, Grealy, & Nosil, 2009; Servedio & Noor, 2003; Smadja & Ganem, 2005). Reinforcing selection often causes reproductive character displacement, increasing prezygotic isolation in sympatry between populations with partial postzygotic isolation (Butlin, 1987; Hopkins et al., 2012; Ortiz‐Barrientos et al., 2009; Servedio & Noor, 2003). During fieldwork, we did not find any hybrids in either sympatric or allopatric populations. Our field experiments also showed that the two varieties are partially isolated postzygotically, which was evident from the much lower seed set in artificial hybridization between the two varieties than that in open pollination, artificial crossing, and selfing within varieties (our unpublished data). Reinforcement selection is assumed not only to complete speciation between two incipient species, but also to initiate speciation by causing the evolution of prezygotic isolation between populations of the species undergoing reinforcement (Higgie & Blows, 2007; Hoskin, Higgie, McDonald, & Mortiz, 2005; Howard, 1993; Lemmon, 2009; Rice & Pfennig, 2010). When reinforcement causes reproductive character displacement, there is divergent selection within a species for different mating signals or mating preferences in different parts of the range (i.e., in allopatry vs. sympatry) (Hopkins et al., 2012). This difference in mating traits can lead to further segregation of sympatric heterospecifics and reduce gene flow between sympatric and allopatric populations of conspecifics (Ortiz‐Barrientos et al., 2009; Pfennig & Pfennig, 2009). We suggest that the two varieties of *O. benthamii* may have initially diverged and acquired sterility barriers in allopatry, followed by a period of range expansion causing secondary contact and reinforcing selection (e.g., flower color alteration—reproductive character displacement) leading to the increased genetic divergence observed in sympatry. However, a more extensive survey combining morphological, ecological, and genomic data could make it possible to reconstruct the phylogeographic history of *O. benthamii* in order to test this hypothesis.

## CONFLICT OF INTEREST

None declared.

## AUTHOR CONTRIBUTIONS

**Qiong Fu:** Conceptualization (supporting); data curation (supporting); formal analysis (supporting); investigation (supporting); methodology (equal); project administration (supporting); software (equal); validation (supporting); visualization (equal); writing – original draft (equal); writing – review & editing (equal). **Guo‐Hui Lu:** Investigation (supporting). **Yu‐Hui Fu:** Investigation (supporting). **Ying‐Qiang Wang:** Conceptualization (lead); data curation (lead); formal analysis (lead); funding acquisition (lead); investigation (lead); methodology (lead); project administration (lead); resources (lead); software (lead); supervision (lead); validation (lead); visualization (lead); writing – original draft (lead); writing – review & editing (lead).

## Supporting information

Supplementary Material

## Data Availability

Inter Simple Sequence Repeat (ISSR) data for two varieties of *Oreocharis benthamii* is available from the Dryad Digital Repository at https://doi.org/10.5061/dryad.jq2bvq86t.
