# Supplementary material for: Genetic differentiation between two varieties of Oreocharis benthamii (Gesneriaceae) in sympatric and allopatric regions
Source: Ecol Evol. 2020 Jun 28;10(14):7792–805. doi: 10.1002/ece3.6505 (PMC7391312; doi:10.1002/ece3.6505)
Supplement: Supplementary file 1 — Supplementary Material [file ECE3-10-7792-s001.docx]

**Supplementary Material**

**TABLE S1.** Details of populations of *Oreocharis benthamii* var. *reticulata* and var. *benthamii* sampled.

| Population | Location | Latitude (N) | Longitude (E) | Altitude (m) | Sample size | Vouchers |
| --- | --- | --- | --- | --- | --- | --- |
| var. *reticulata* | |  |  |  |  |  |
| SDH1 | Dinghu Mountain, Zhaoqing, Guangdong  Dinghu Mountain, Zhaoqing, Guangdong  Dinghu Mountain, Zhaoqing, Guangdong | 23º10.189' | 112º28.436' | 450 | 30 | TGB-4 |
| SDH2 |  | 23º10.542' | 112º32.285' | 477 | 28 | GYF-44 |
| SDH3 |  | 23º02.832' | 112º27.905' | 440 | 30 |  |
| SHYB | Nanca, Jiaoling, Guangdong | 24º45.591' | 116º16.454' | 591 | 29 | WBC-221 |
| SCT | Changtan, Jiaoling, Guangdong | 24º43.315' | 116º07.998' | 248 | 29 | WBC-222 |
| SYN | Yinna, Meixian, Guangdong | 24º24.382' | 116º24.209' | 582 | 29 | WBC-226 |
| SXTS1* | Xiangtou Mountain, Boluo, Guangdong | 23º16.798' | 114º22.130' | 700 | 30 | WBC-166 |
| SXTS2* | Xiangtou Mountain, Boluo, Guangdong | 23º16.498' | 114º22.364' | 647 | 30 | WBC-168 |
| SXMS | Longmen, Nanning, Guangxi | 22º51.181' | 107º40.181' | 946 | 30 | WBC-214 |
| SDMS | Nalong, Congzuo, Guangxi | 23º26.274' | 108º18.638' | 1000 | 26 | GYF-123 |
| SJX | Tongmu, Jinxiu, Guangxi | 24º09.429' | 110º06.738' | 1034 | 27 | WBC-218 |
| SDGS | Babu, Hezhou, Guangxi | 24º09.330' | 111º42.420' | 220 | 30 | FQ2015-16 |
| SSGZ | Nabu, Bobai, Guangxi | 21º53.200' | 109º49.472' | 570 | 30 | FQ2015-06 |
| SBYS | Linfeng, Hezhou, Guangxi | 24º01.482' | 110º54.280' | 145 | 30 | FQ2015-12 |
| SWY | Zhankeng, Shaoguan, Guangdong | 24º22.886' | 113º52.439' | 191 | 30 | FQ2016-14 |
| SQY | Xiutianluo, Qingyuan, Guangdong | 23º46.301' | 113º08.758' | 122 | 30 | FQ2016-06 |
| SMB | Daxi, Xinfeng, Guangdong | 24º12.917' | 114º31.917' | 360 | 30 | FQ2016-16 |
| SCZ | Jiaoshuikeng, Chaozhou, Guangdong | 23º51.265' | 116º44.459' | 610 | 30 | WYQ2017-01 |
| total |  |  |  |  | 528 |  |
| var. *benthamii* | |  |  |  |  |  |
| DXTS1* | Xiangtou Mountain, Boluo, Guangdong  Xiangtou Mountain, Boluo, Guangdong  Xiangtou Mountain, Boluo, Guangdong  Xiangtou Mountain, Boluo, Guangdong | 23º17.058' | 114º20.700' | 589 | 30 | GYF-45 |
| DXTS2* |  | 23º16.634' | 114º20.750' | 305 | 29 | WBC-172 |
| DXTS3* |  | 23º17.414' | 114º21.782' | 711 | 30 | WBC-165 |
| DXTS4* |  | 23º19.173' | 114º19.527' | 550 | 18 | WBC-242 |
| DFX | Fengxi, Dapu, Guangdong | 24º38.802' | 116º47.017' | 396 | 30 | WBC-227 |
| DNJ | Daling, Najing, Fujian | 24º31.069' | 117º12.419' | 400 | 29 | WBC-243 |
| DLH | Nanwan, Luhe, Guangdong | 23º21.067' | 115º30.767' | 419 | 30 | WYQ2017-06 |
| DBX | Baixi, Zijin, Guangdong | 23º44.867' | 115º16.017' | 558 | 30 | WYQ2017-87 |
| total |  |  |  |  | 226 |  |
| Species | |  |  |  | 754 |  |

*-- populations in sympatry; other populations in allapatry.

**TABLE S2.** Attributes of ten ISSR primers used in the present study

| Primer | Sequence 5´to 3´ | *T*m / ℃ | SR | NT | NP |
| --- | --- | --- | --- | --- | --- |
| 808 | (AG)_8_C | 46 | 320-1800 | 44 | 44 |
| 834 | (AG)_8_Y*T | 56 | 160-1700 | 51 | 51 |
| 841 | (GA)_8_Y*C | 59 | 120-2200 | 56 | 56 |
| 847 | (CA)_8_R*C | 58 | 240-2200 | 46 | 46 |
| 857 | (AC)_8_Y*G | 46 | 220-1800 | 38 | 38 |
| 873 | (GACA)_4_ | 48 | 300-1800 | 39 | 39 |
| 879 | (CTTCA)_3_ | 50 | 250-2000 | 49 | 49 |
| 881 | GGG(TGGGG)_2_TG | 54 | 290-2200 | 43 | 43 |
| 899 | CATGGTGTTGGTCATTGTTCCA | 56 | 300-2000 | 44 | 44 |
| 900 | ACTTCCCCACAGGTTAACACA | 52 | 200-2000 | 44 | 44 |
| Total | - | - | - | 454 | 454 |

*Y: C/T, R: A/G;

*T*m: annealing temperature; SR: size range of amplified fragments; NT: number of total bands; NP: number of polymorphic bands;

**TABLE** **S3.** Estimation of the most likely number of populations presented in the sample of *Oreocharis benthamii* based on the Bayesian method implemented in STRUCTURE

| K | Mean Pr[X\|K] | ΔK | Parsimony Index |
| --- | --- | --- | --- |
| 1 | -142929.22 | - | 0.5000 |
| 2 | -129003.46 | **1943.5561** | 0.7528 |
| 3 | -121144.86 | 2.2244 | 0.6969 |
| 4 | -114393.93 | 2.1684 | 0.7382 |
| 5 | -108557.24 | 0.9614 | 0.8242 |
| 6 | -103480.06 | 1.1438 | 0.9253 |
| 7 | -99724.45 | 1.0866 | 0.7757 |
| 8 | -94364.54 | 1.5040 | 0.8687 |
| 9 | -91562.65 | 0.4022 | 0.8482 |
| 10 | -89431.34 | 0.4600 | 0.8383 |
| 11 | -86203.12 | 1.8195 | 0.9011 |
| 12 | -86157.04 | 0.6391 | 0.8692 |
| 13 | -79667.41 | 1.3690 | 0.8892 |
| 14 | -79440.97 | 0.7395 | 0.9236 |
| 15 | -76419.97 | 11.1782 | **0.9420** |
| 16 | -96710.55 | 0.7491 | 0.8728 |
| 17 | **-70207.48** | 73.5258 | 0.9141 |
| 18 | -185239.03 | 0.5033 | 0.8760 |
| 19 | -161717.08 | 0.0792 | 0.8987 |
| 20 | -152263.92 | 0.5095 | 0.9130 |
| 21 | -209642.53 | 0.3587 | 0.9187 |
| 22 | -164150.66 | 0.2184 | 0.8936 |
| 23 | -162332.78 | 0.1077 | 0.8505 |
| 24 | -179903.96 | 2.9060 | 0.8805 |
| 25 | -521565.03 | 0.9091 | 0.8565 |
| 26 | -493231.58 | - | 0.8531 |

**
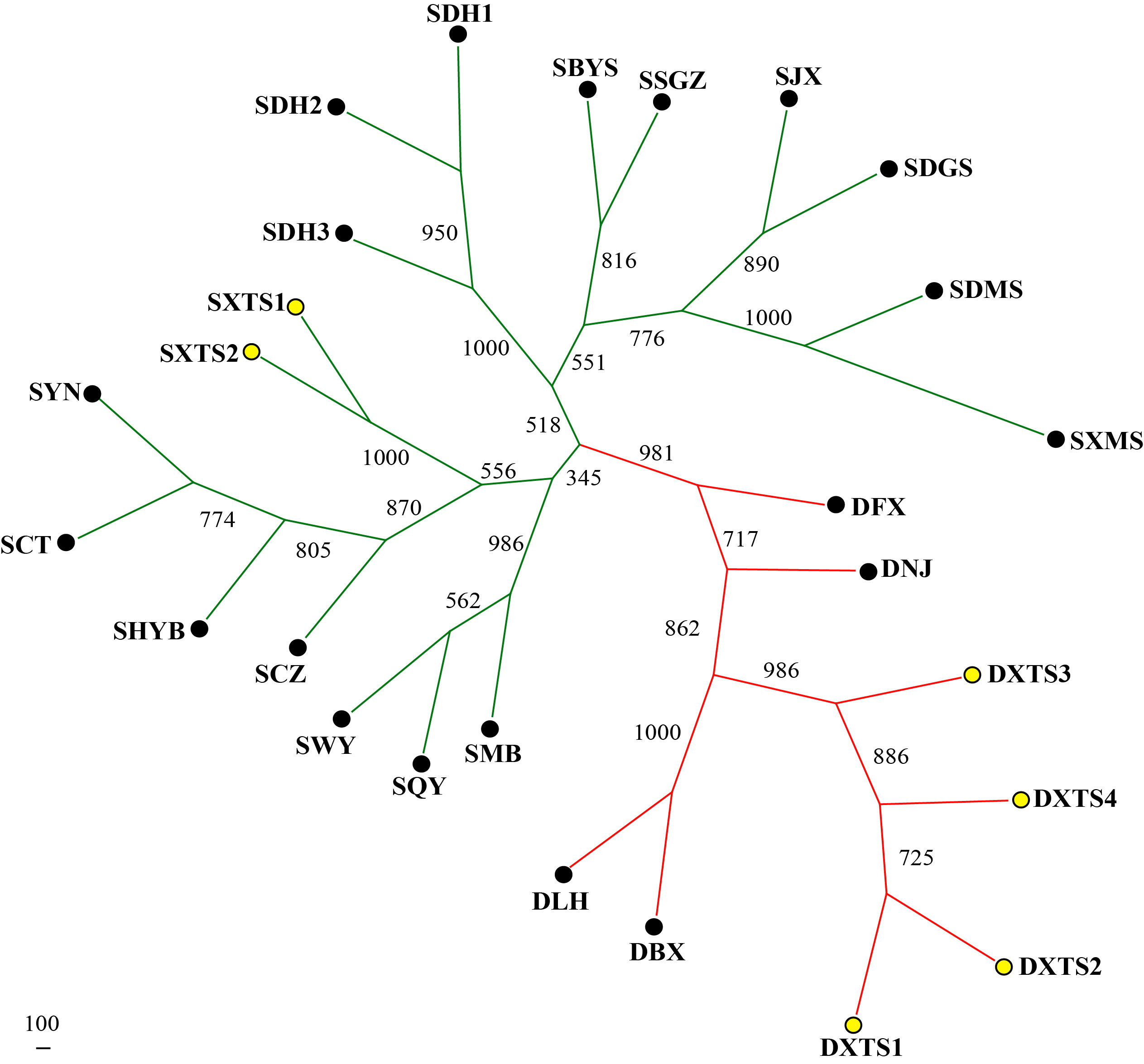
**

**FIGURE** **S1.** Unrooted neighbor-joining (NJ) tree based on Nei’s genetic distance for 26 populations of *Oreocharis benthamii* var. *reticulata* (green) and var. *benthamii* (red); black circles indicate allopatric populations, yellow circles indicate sympatric populations.

**
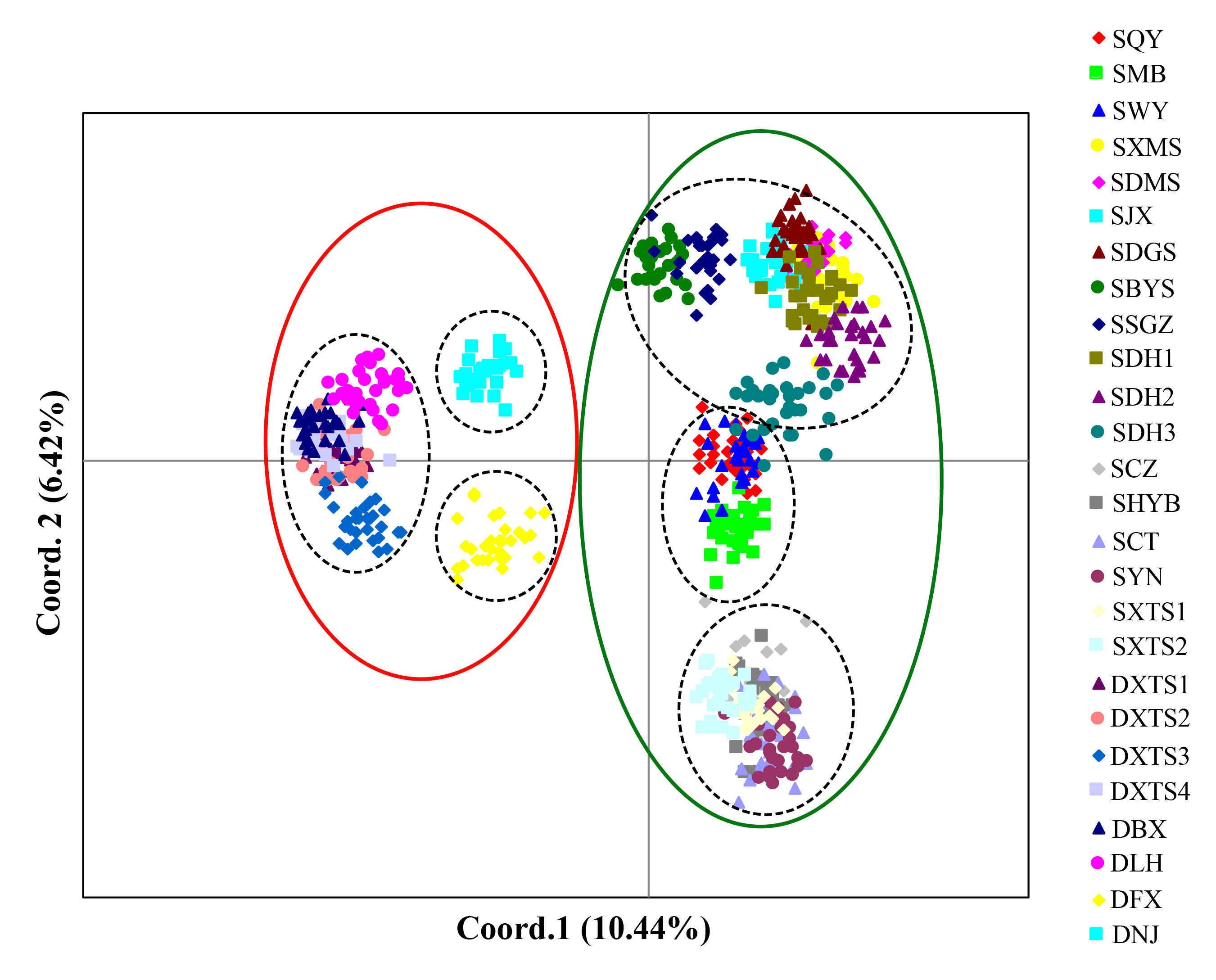
**

**FIGURE S2**. Scatterplot of the principal coordinate analysis (PCoA) based on ISSR polymorphisms of 754 samples from 26 populations of *Oreocharis benthamii* var. *reticulata* (green solid circle) and var. *benthamii* (red solid circle).
